# High Serum S100A12 as a Diagnostic and Prognostic Biomarker for Severity, Multidrug-Resistant Bacteria Superinfection and Herpes Simplex Virus Reactivation in COVID-19

**DOI:** 10.3390/v16071084

**Published:** 2024-07-05

**Authors:** Patricia Mester, Dennis Keller, Claudia Kunst, Ulrich Räth, Sophia Rusch, Stephan Schmid, Sabrina Krautbauer, Martina Müller, Christa Buechler, Vlad Pavel

**Affiliations:** 1 Department of Internal Medicine I, Gastroenterology, Hepatology, Endocrinology, Rheumatology, and Infectious Diseases, University Hospital Regensburg, 93053 Regensburg, Germany; patricia.mester@klinik.uni-regensburg.de (P.M.); dennis.keller@stud.uni-regensburg.de (D.K.); claudia.kunst@klinik.uni-regensburg.de (C.K.); ulrich.raeth@stud.uni-regensburg.de (U.R.); sophia.rusch@klinik.uni-regensburg.de (S.R.); stephan.schmid@klinik.uni-regensburg.de (S.S.); martina.mueller-schilling@klinik.uni-regensburg.de (M.M.); vlad.pavel@klinik.uni-regensburg.de (V.P.); 2 Institute of Clinical Chemistry and Laboratory Medicine, University Hospital Regensburg, 93053 Regensburg, Germany; sabrina.krautbauer@klinik.uni-regensburg.de

**Keywords:** SARS-CoV-2, herpes simplex, vancomycin resistance, neutrophils, S100A12

## Abstract

Neutrophils are critical immune cells in severe coronavirus disease 2019 (COVID-19). S100 calcium-binding protein A12 (S100A12) is highly expressed in neutrophils during acute inflammation. The aim of this study was to evaluate serum S100A12 levels as a diagnostic and prognostic tool in COVID-19. Serum samples of patients with moderate and severe COVID-19 were collected during 2020 to 2024. Enzyme-linked immunosorbent assay was used to measure serum S100A12 levels in 63 patients with moderate COVID-19, 60 patients with severe disease and 33 healthy controls. Serum S100A12 levels were elevated in moderate COVID-19 compared to controls and were even higher in severe cases. In moderate disease, serum S100A12 levels positively correlated with immune cell counts. While C-reactive protein and procalcitonin are established inflammation markers, they did not correlate with serum S100A12 levels in either patient cohort. Patients with severe COVID-19 and vancomycin-resistant enterococcus (VRE) infection had increased S100A12 levels. Elevated S100A12 levels were also observed in patients with herpes simplex reactivation. Fungal superinfections did not alter S100A12 levels. These data show that serum S100A12 increases in moderate and severe COVID-19 and is further elevated by VRE bloodstream infection and herpes simplex reactivation. Therefore, S100A12 may serve as a novel biomarker for severe COVID-19 and an early diagnostic indicator for bacterial and viral infections.

## 1. Introduction

Severe acute respiratory syndrome coronavirus 2 (SARS-CoV-2) was first reported in December 2019, and led to a global COVID-19 pandemic [1,2]. Recent studies have reported a significant increase in the neutrophil counts in patients with COVID-19. Patients with severe COVID-19 had significantly higher neutrophil counts on admission compared to patients with mild or moderate disease. Neutrophilia was associated with disease severity and poor prognosis [3,4].

S100 calcium-binding protein A12 (S100A12, also called calgranulin C) is mainly expressed by neutrophils [5,6]. Binding of calcium triggers its translocation from the cytosol to the membrane [6]. Extracellular S100A12 acts as a chemoattractant for monocytes and mast cells and activates pathways that increase cytokine production and oxidative stress [7,8]. The receptor for advanced glycation endproducts (RAGE) is the best known receptor for S100A12 and binding of this ligand activates nuclear factor kappa B and mitogen-activated protein kinase to induce the expression of proinflammatory cytokines [6,9]. Previous studies have also shown antifungal and antibacterial activities of S100A12. Copper and zinc sequestration by S100A12 and membrane permeabilisation caused by the phospholipid binding capacity of S100A12 confers antimicrobial activity [10,11]. Knockdown of S100A12 in macrophages reduced the toll-like receptor 2 and interferon-gamma response to *Mycobacterium leprae*, resulting in decreased pathogen killing, illustrating the protective effect of this protein [12]. S100A12 and CD177 gene-expression analysis in blood effectively diagnosed bacterial infections with high sensitivity and specificity. This gene-expression analysis outperformed serum procalcitonin and C-reactive protein in diagnosing bloodstream infections [13]. 

Serum S100A12 levels are increased in infectious and non-infectious inflammatory diseases such as inflammatory bowel disease, rheumatoid arthritis, sepsis and community-acquired pneumonia [6,8,14]. Serum S100A12 has been shown to be elevated in acute otitis media caused by *Streptococcus pneumoniae* and nontypeable *Haemophilus influenzae* but not in viral upper respiratory tract infections [15]. Serum S100A12 was also associated with disease severity and survival of patients with community-acquired pneumonia, which is mostly caused by *Streptococcus pneumoniae* [14]. 

Furthermore, in patients with respiratory distress syndrome higher levels of S100A12 have been detected in bronchoalveolar fluid [16].

The role of S100A12 in SARS-CoV-2 infection is not well understood. Transcriptomic analysis of whole blood and peripheral blood mononuclear cells from COVID-19 patients showed S100A12 activation primarily in severe cases [17]. S100A12 expression was also elevated in the lung tissues of COVID-19 patients [18]. Studies measuring circulating S100A12 in COVID-19 are sparse. One study reported higher S100A12 levels in COVID-19 patients compared to healthy controls, with further increases in severe cases and a correlation with mortality [19]. 

Our study aimed to measure serum S100A12 levels in controls and patients with moderate and severe COVID-19 to assess associations with disease severity. Bacterial superinfections are known risk factors for worse COVID-19 outcomes, yet early diagnostic markers are lacking [20,21]. We therefore also investigated whether serum S100A12 levels increase in patients with bacterial and fungal superinfections or herpes simplex virus (HSV) reactivation, which are associated with more severe COVID-19 disease [22]. 

## 2. Materials and Methods

### 2.1. Study Cohort

Blood samples were collected from adult patients with confirmed SARS-CoV-2 infection between April 2020 and January 2024. The study was performed in line with the Helsinki Declaration and approved by the Ethics Committee of the University Hospital of Regensburg (protocol code 18-1029_2-101, 14 March 2023). All participants gave their written consent. Blood of patients with moderate disease was collected 3 (1–16) days after hospital admission, and of patients with severe COVID-19 4 (1–10) days after hospital admission. Serum S100A12 did not correlate with the day of blood collection in the moderate (r = −0.088, *p* = 0.596) and severe (r = 0.202, *p* = 0.122) group. In the moderate and the severe cohort, serum CRP, procalcitonin, lactate dehydrogenase, ferritin, neutrophil count, basophil and immature granulocytes number did not correlate with the day of blood collection, showing that the day of blood collection was not associated with these measures of disease severity.

In Germany, the first vaccines against SARS-CoV-2 were administered on 26 December 2020, and most of our patients had not yet completed vaccination. Patients with COVID-19 received treatment following the European Medicines Agency and German Federal Joint Committee guidelines. In Germany, COVID-19 treatment included remdesivir and dexamethasone, with heparin administered to all patients to prevent blood clots.

Sixty-three patients had fever, tachycardia, dyspnoea and fatigue. These patients fulfilled the criteria for systemic inflammatory response syndrome (SIRS) and were assigned to the “moderate” COVID-19 group [23,24]. This group also fulfilled the National Institutes of Health (NIH) criteria for moderate disease [25]. The patients were hospitalised but did not need to be admitted to the intensive care unit. Sixty patients developed septic shock and almost all acute respiratory distress syndrome and were treated in the intensive care unit. Our “severe” group of patients corresponds to critical illness, according to the NIH classification of COVID-19 severity [24,25,26,27]. 

### 2.2. Measurement of Serum S100A12 

Serum S100A12 levels were measured using the IDK^®^ S100A12 ELISA kit (Immundiagnostik AG, Bensheim, Germany). Each sample was tested in duplicate, and the average result was used. For the ELISA test, a 1:40 dilution of each sample was prepared.

### 2.3. Microbiological Tests

Blood cultures and Gram staining were conducted at the Institute of Clinical Microbiology and Hygiene, University Hospital Regensburg. Bacteria and fungi were identified using MALDI-TOF mass spectrometry. Antimicrobial susceptibility was determined by minimum inhibitory concentration measurements according to EUCAST guidelines or automated systems. Vancomycin resistance in enterococci was confirmed by PCR detection of van A and/or van B genes. Herpes simplex virus was detected in bronchoalveolar lavage samples using PCR.

### 2.4. Statistical Analysis

All figures show data as boxplots, with the minimum value, the maximum value, the median and the first and third quartiles. Outliers are indicated by circles (S100A12 levels >1.5 × the interquartile range) and asterisks (S100A12 levels >3.0 × the interquartile range). Table data presents the median, minimum and maximum values. We used IBM SPSS Statistics 26.0 to analyse the data. Kolmogorov–Smirnov test and the Shapiro–Wilk test showed that the data were not normally distributed (*p* < 0.001 for both tests), and therefore, non-parametric statistical tests were used. We applied the Chi-Square test, Receiver Operating Characteristics Curve, Mann–Whitney U test, Kruskal–Wallis test and Spearman’s correlation to test for significance. We considered a *p*-value < 0.05 to be significant.

## 3. Results

### 3.1. Serum S100A12 Levels of Healthy Controls and Patients with Moderate and Severe COVID-19 Disease 

S100A12 protein levels were measured in the serum of 33 controls, 63 patients with moderate and 60 patients with severe COVID-19. Controls and patients had similar sex distribution and age (Table 1). C-reactive protein (CRP), procalcitonin, lactate dehydrogenase (LDH) and ferritin levels were higher in severe compared to moderate disease (Table 1). Age, sex, alkaline phosphatase (AP) and interleukin-6 were similar between the two groups (Table 1). 

Patients with severe COVID-19 had a higher body mass index (BMI) and increased levels of neutrophils, basophils, monocytes and immature granulocytes. Eosinophil and lymphocyte counts did not differ between the groups. Viral load was similar in both cohorts, but SARS-CoV-2 antibody titers were significantly higher in patients with severe COVID-19 (Table 1).

**Figure 1:**
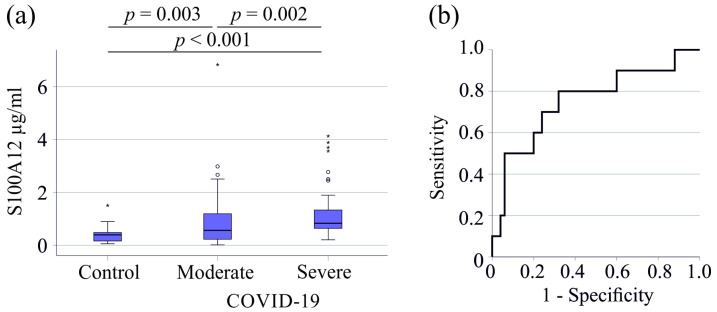
Serum S100A12 levels of healthy controls and patients with COVID-19. (**a**) Serum S100A12 levels of controls, patients with moderate and severe COVID-19; (**b**) receiver operating characteristic curve for discrimination of moderate and severe COVID-19. Outliers are indicated by circles (S100A12 levels >1.5 × the interquartile range) and asterisks (S100A12 levels >3.0 × the interquartile range).

Serum S100A12 levels of controls were the lowest with 0.39 (0.06–1.51) µg/mL, were higher in moderate COVID-19 with 0.56 (0.02–6.81) µg/mL and with 0.83 (0.21–4.13) µg/mL were highest in severe COVID-19 patients (Figure 1a).

Serum S100A12 had an area under the receiver operating characteristic curve (AUROC) for predicting severe compared to moderate COVID-19 of 0.643 (*p* = 0.006), indicating that it is not an excellent marker for assessing disease severity (Figure 1b).

### 3.2. Serum S100A12 Levels in Relation to Sex, Age and BMI 

Male and female controls had similar serum S100A12 levels (*p* = 0.931), with no correlation to age (r = −0.276, *p* = 0.239). In the moderate COVID-19 group, serum S100A12 levels did not correlate with age (r = −0.197, *p* = 0.125) or BMI (r = −0.110, *p* = 0.547) and were similar between sexes (*p* = 0.258). In severe COVID-19, females tended to have lower serum S100A12 levels compared to males (*p* = 0.060). Serum S100A12 was not related to age (r = 0.188, *p* = 0.154) or BMI (r = −0.072, *p* = 0.602) in this group as well.

### 3.3. Serum S100A12 Levels with Viral Load and Antibody Titer

Serum S100A12 did not correlate with the viral load in patients with moderate (r = 0.117, *p* = 0.392) and severe (r = 0.076, *p* = 0.607) COVID-19 and also did not correlate with antibody titer in moderate (r = 0.188, *p* = 0.603) and severe (r = 0.046, *p* = 0.752) disease.

### 3.4. Serum S100A12 in COVID-19 Patients Receiving Dialysis and Vasopressor Therapy 

The 6 patients with moderate disease and the 7 patients with severe COVID-19 needing dialysis had similar serum S100A12 levels compared to patients with no need for dialysis (Table 2). Vasopressor therapy of 41 patients with severe COVID-19 was not related to higher S100A12 levels (Table 2). All but one patient were invasively ventilated in the severe COVID-19 cohort. 

### 3.5. Correlation of Serum S100A12 with Inflammation Markers and White Blood Cell Count

Serum S100A12 positively correlated with neutrophils, basophils, monocytes, lymphocytes and immature granulocytes in patients with moderate COVID-19. Such associations did not exist in the severe cohort. CRP, procalcitonin, interleukin-6 and ferritin did not correlate with S100A12 in both patient cohorts (Table 3). 

### 3.6. Serum S100A12 in COVID-19 Patients with Bacterial and Fungal Superinfections and HSV Reactivation

In the cohort with moderate COVID-19, six patients were infected with bacteria, but serum S100A12 of infected and non-infected patients was similar (*p* = 0.100). Two patients were infected with fungi and no patient showed reactivation of HSV. 

**Figure 2:**
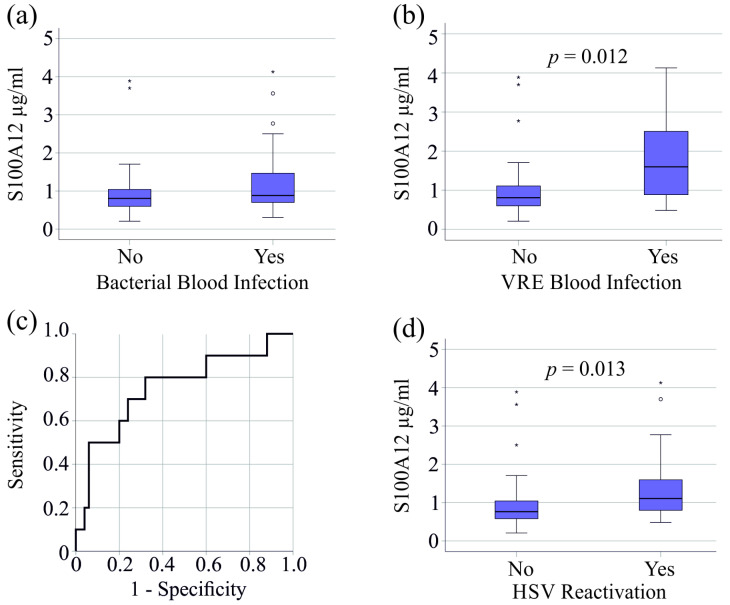
Serum S100A12 levels of patients with severe COVID-19 and bacterial superinfection or herpes simplex virus reactivation. (**a**) Serum S100A12 levels of patients with severe COVID-19 without (No) and with (Yes) bacteremia; (**b**) serum S100A12 levels of patients with severe COVID-19 without (No) and with (Yes) vancomycin-resistant bacteria (VRE) superinfection (**c**) ROC curve for the discrimination of patients with and without vancomycin-resistant bacteria; (**d**) serum S100A12 levels of patients with severe COVID-19 with (Yes) and without (No) herpes simplex virus (HSV) reactivation. Outliers are indicated by circles (S100A12 levels >1.5 × the interquartile range) and asterisks (S100A12 levels >3.0 × the interquartile range).

In the patient cohort with severe COVID-19, the 27 patients with bacterial bloodstream infections had similar serum S100A12 levels compared to non-infected patients (*p* = 0.136) (Figure 2a). It is noteworthy that 10 patients with bloodstream infection caused by vancomycin-resistant enterococci (VRE) exhibited higher serum S100A12 levels (*p* = 0.012) (Figure 2b). The AUROC of serum S100A12 to discriminate patients with and without VRE was 0.754 (*p* = 0.012) (Figure 2c). A concentration of 0.88 µg/mL S100A12 exhibited an 80% sensitivity and a 68% specificity for the diagnosis of VRE superinfection in patients with severe COVID-19. 

HSV reactivation in 20 patients with severe COVID-19 was related to higher serum S100A12 levels (*p* = 0.013) (Figure 2d). AUROC was 0.695 (*p* = 0.013) and 0.64 µg/mL had a sensitivity of 95% and a specificity of 36% for the diagnosis of HSV reactivation. 

It has to be noted that 6 of our patients with VRE superinfection also had HSV reactivation. The 14 patients with HSV and without VRE had higher serum S100A12 levels compared to patients without HSV reactivation (*p* = 0.027). The 4 VRE patients with VRE and without HSV still had increased serum S100A12 levels (*p* = 0.052). 

The 21 severe COVID-19 patients with fungal superinfection had serum S100A12 comparable to those in the patients without fungal infection (*p* = 0.871, Figure S1). 

CRP and procalcitonin did not change with bacterial, VRE and fungal superinfections or HSV reactivation. 

### 3.7. Serum S100A12 and Survival

**Figure 3:**
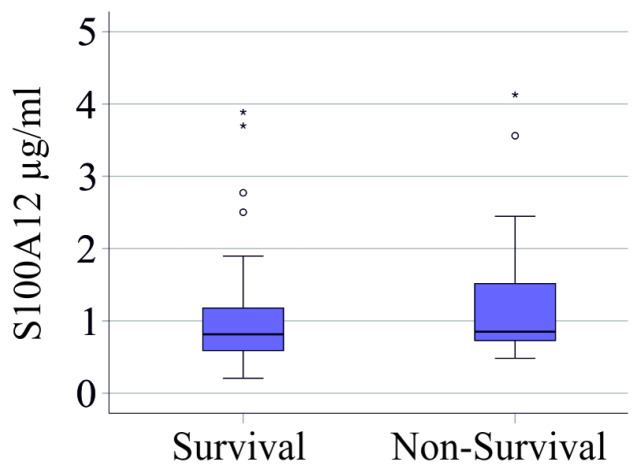
Serum S100A12 and survival. Serum S100A12 levels of surviving and non-surviving patients with severe COVID-19. Outliers are indicated by circles (S100A12 levels >1.5 × the interquartile range) and asterisks (S100A12 levels >3.0 × the interquartile range).

In the patient cohort with severe COVID-19 21 patients died. In patients with severe COVID-19 serum S100A12 of non-survivors and survivors was comparable (*p* = 0.185) (Figure 3). Analysis of the association of serum S100A12 with survival in the whole cohort, including patients with moderate disease, showed a trend to higher levels in non-survivors (*p* = 0.066).

## 4. Discussion

Here we show that serum S100A12 is elevated in SARS-CoV-2 infection and is associated with disease severity, VRE superinfections and HSV reactivation.

For S100A12, data on circulating levels in COVID-19 patients are scarce. Whole blood S100A12 expression in COVID-19 patients has been shown to correlate with disease severity and outcome [28]. Murphy et. al. described an increase in circulating S100A12 in COVID-19, which was even higher in severe cases. Elevated S100A12 levels persisted during the 10-day follow-up [19]. The current analysis is in accordance with this previous study. Serum S100A12 levels of moderate COVID-19 patients were higher in comparison to healthy controls and further increased in severe cases.

This previous analysis observed higher mortality of patients with elevated S100A12 levels [19]. In accordance with these data our analyses of the association of serum S100A12 with survival in the whole cohort, including patients with moderate disease, showed a trend to higher levels in non-survivors [19].

S100A12 was described to function as an antimicrobial peptide for bacterial and fungal infections [11,12,29]. Serum levels of S100A12 were induced in patients with bacterial infections and patients with sepsis [8,15]. We show here that serum S100A12 levels were increased in patients with VRE bloodstream infections. To the best of our knowledge, this finding has not been described before.

Among COVID-19 patients with bacterial superinfections, the proportion of enterococci infections that were resistant to vancomycin has been shown to be around 19% and there was substantial heterogeneity in the different studies [30]. In patients with severe COVID-19 in our cohort, 37% of bacterial bloodstream infections were VRE. These latter patients had elevated serum S100A12 levels. A serum concentration of 0.88 µg/mL S100A12 indicates VRE with an 80% sensitivity and a 68% specificity in patients with severe COVID-19.

HSV is a common virus, with a global prevalence of 67% for HSV1 and 13% for HSV2 [31]. Reactivation of HSV is frequent in patients with COVID-19 who require prolonged invasive mechanical ventilation [32], and almost all of our patients with severe COVID-19 were invasively ventilated. In our study cohort serum S100A12 was significantly increased in patients with HSV reactivation. To the authors’ knowledge this fact has not been described before.

Reactivation of varicella-zoster virus has also been noted in COVID-19 [33] but this was not the case in our patient cohort. Moreover, the reactivation of varicella-zoster virus has been observed after COVID-19 vaccination [34]. However, a multicentre observational cohort study could not observe an association between varicella-zoster caused neurologic disease and COVID-19 vaccination [35]. Thus, the association of COVID-19 vaccination, SARS-CoV-2 infection and reactivation of varicella-zoster virus needs further study.

S100A12 was described to induce proinflammatory responses in different immune cells [5,6,7,8]. Serum S100A12 did not correlate with CRP, procalcitonin and interleukin-6 levels in the serum of patients with moderate and severe COVID-19. This shows that S100A12 is not associated with these established markers of inflammation. Notably, serum S100A12 correlated positively with the number of neutrophils, likely because this protein is mainly released by these cells [6]. Moreover, there were positive correlations of serum S100A12 with basophils, monocytes, lymphocytes and immature granulocytes in moderate COVID-19. In severe COVID-19, there was no association between serum S100A12 and these immune cells. The neutrophil count was approximately 2-fold higher in severe compared to moderate COVID-19, while the increase in serum S100A12 was 1.5-fold, suggesting that S100A12 production by neutrophils is impaired in severe disease. Consistent with our data neutrophil dysfunction in patients with COVID-19 increases with disease progression [36,37,38].

Patients with severe COVID-19 disease had more neutrophils, basophils, monocytes and immature granulocytes in blood. Elevated neutrophils in severe COVID-19 disease have been described before. However, basophils and monocytes were also found to decline with higher disease severity [4,39]. Further studies could not detect significant differences in the number of monocytes between patients with COVID-19 and controls and reported the expansion of specific monocyte subpopulations in severe COVID-19 [40]. Further analysis is needed to resolve the changes in the number and function of immune cells in patients with SARS-CoV-2 infections.

Compared to patients with moderate COVID-19 in our study, those with severe disease had significantly higher SARS-CoV-2 antibody titers, consistent with previous studies [41,42]. However, serum S100A12 levels did not correlate with antibody titer or viral load.

Age, BMI and sex are confounding factors in clinical studies. Serum S100A12 did not correlate with BMI and age of controls and COVID-19 patients and did not significantly differ between sexes. This shows that these variables do not greatly affect circulating levels of S100A12.

This study has limitations. The laboratory values of the controls were not determined. All our controls were healthy and had normal body weight, suggesting that the laboratory values were in the normal range. All our patients and controls were from Bavaria/Germany and the results may not apply to patients from other countries. The number of patients with VRE infection was small and data need to be confirmed in further multicentre studies.

## 5. Conclusions

This analysis identified serum S100A12 levels as a marker of severe COVID-19. Our study is the first to demonstrate the potential of serum S100A12 in identifying patients with VRE bloodstream superinfection and HSV reactivation. If confirmed in larger studies, serological measurement of S100A12 could improve antibiotic and antiviral drug selection for COVID-19 patients.

## Figures and Tables

**Table 1:** Characteristics of patients and controls (alkaline phosphatase: AP; arbitrary unit: AU; body mass index: BMI; lactate dehydrogenase: LDH). Superscript numbers indicate that laboratory values were not documented for the entire group of patients, but for a subset of patients. The *p*-values are given in the table.

Parameter	Moderate COVID-19	Severe COVID-19	Controls
Males/Females	34/29	42/18	15/18
Age (years)	60 (22–83)	57 (31–83)	56 (50–81)
BMI (kg/m^2^)	26.3 (18.4–42.6)^32^	29.4 (19.2–66.7)^56; *p* = 0.008^	Not determined
C-reactive protein mg/L	26 (0–222)	74 (1–367) *^p^* ^< 0.001^	Not determined
Procalcitonin ng/mL	0.09 (0–24.90)	0.24 (0.06–25.00)^*p* < 0.001^	Not determined
LDH U/L	224 (127–929)^39^	378 (162–1534)^*p* < 0.001^	Not determined
AP U/L	96 (38–372)^29^	99 (37–743)	Not determined
Ferritin ng/mL	573 (32–4826)^45^	1088 (77–21976)^60; *p* < 0.001^	Not determined
Interleukin-6 pg/mL	19 (4–265)^37^	36 (3–1175)	Not determined
Neutrophils n/nL	4.05 (0.13–23.10)	8.18 (0.90–24.91)^*p* < 0.001^	Not determined
Basophils n/nL	0.03 (0–0.21)	0.05 (0.01–0.17) *^p^* ^< 0.001^	Not determined
Eosinophils n/nL	0.08 (0–1.19)	0.04 (0–1.07)	Not determined
Monocytes n/nL	0.57 (0.07–2.52)	0.71 (0.03–2.21) *^p^* ^= 0.037^	Not determined
Lymphocytes n/nL	1.11 (0.09–57.83)	1.20 (0–75.95)	Not determined
Immature Granulocytes n/nL	0.03 (0–1.38)	0.25 (0.04–2.92) ^*p* < 0.001^	Not determined
Viral Load	8600 (48–19 × 10^6^)^57^	14,000 (95–52 × 10^7^)^49^	Not determined
Antibody AU/mL	101 (14–1487)^10^	661 (17–1939)^50; *p* = 0.034^	Not determined

**Table 2:** Serum S100A12 levels (µg/mL) of patients on dialysis and vasopressor therapy in comparison to patients without this intervention/therapy.

Intervention/Drug	No	Yes
Moderate COVID-19		
Dialysis (6 patients)	0.60 (0.02–2.98)	0.33 (0.15–6.83)
Severe COVID-19		
Dialysis (7 patients)	0.81 (0.21–3.89)	1.11 (0.62–4.13)
Catecholamine (41 patients)	0.86 (0.35–2.50)	0.80 (0.21–4.13)

**Table 3:** Spearman correlation coefficients for the correlation of serum S100A12 levels with inflammatory parameters and immune cell counts.

Inflammation Marker	Moderate COVID-19	Severe COVID-19
C-reactive Protein	0.207	0.060
Procalcitonin	0.112	0.149
Interleukin-6	−0.065	0.006
Ferritin	0.151	0.216
Neutrophils	0.525 *^p^* ^< 0.001^	0.179
Basophils	0.293 *^p^* ^= 0.002^	0.030
Eosinophils	0.189	−0.046
Monocytes	0.368 *^p^* ^= 0.003^	−0.125
Lymphocytes	0.306 *^p^* ^= 0.011^	0.011
Immature Granulocytes	0.489 *^p^* ^< 0.001^	0.101

## Data Availability

The data supporting the findings of this study are available within the article.

## Supplementary Materials

The following supporting information can be downloaded at: https://www.mdpi.com/article/10.3390/v16071084/s1, Figure S1: Serum S100A12 levels of patients with severe COVID-19 and fungal superinfections.
