# Supplementary figures and images for: High Serum S100A12 as a Diagnostic and Prognostic Biomarker for Severity, Multidrug-Resistant Bacteria Superinfection and Herpes Simplex Virus Reactivation in COVID-19
Source: Viruses. 2024 Jul 5;16(7):1084. doi: 10.3390/v16071084 (PMC11281500; doi:10.3390/v16071084)

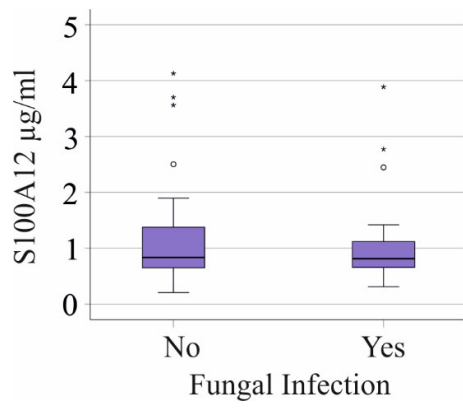

**Figure S1.** Serum S100A12 levels of patients with severe COVID-19 and fungal superinfections.

Supplement: Supplementary file 1 [file viruses-16-01084-s001.zip › viruses-3080494-supplementary.pdf]
